# Osteoarthritis in a symptomatic coracoclavicular joint

**DOI:** 10.15761/gmt.1000106

**Published:** 2018-03-30

**Authors:** Alexander Schuh, Frank Seehaus, Ndubuisi OC Onyemaechi, Wolfgang Hönle

**Affiliations:** 1 Musculoskeletal Center, Neumarkt Hospital, 92318 Neumarkt, Germany; 2 Orthopaedic Department University of Erlangen-Nuremberg, Germany; 3 Department of Surgery, University of Nigeria Teaching Hospital, Ituku-Ozalla Enugu, Nigeria

## Abstract

Coracoclavicular joint (CCJ) is a rare cause of shoulder pain. CCJ is not described in most orthopaedic textbooks, leading to lack of awareness in the general orthopaedic community. In that way the incidence of symptomatic cases is underestimated. We present the case of a symptomatic osteoarthtritic CCJ in a 46-year-old male patient with nearly complete relief of pain after therapeutic injection of the CCJ. The radiological signs of CCJ are briefly discussed to increase awareness of this very rare entity.

## Introduction

CCJ is a rare anomalous joint found between the coracoid process of the scapula and the conoid tubercle of the clavicle A true synovial diarthroidal coracoclavicular joint in humans is rare. Gruber was first to describe the entity in 1861 [1]. Prevalance is depending upon the mode of investigation and the population sample. A wide variation has been published ranging from 0.7% to 10% in osteological studies, 1.7% to 30% in cadaveric dissections and 0.04% to 3.0% in radiological studies [1–6]. Singh V *et al.* [1] analysed all published cases about symptomatic CCJ. In their thorough analysis of 21 symptomatic CCJ the most common presenting feature was shoulder pain in 12 (70.58%) patients, followed by limitation of shoulder movements associated with painful arc in five patients (29.41%). The other associated complaints were upper-limb paresthesia in 4 (23.53%) cases, brachialgia and radiation pain to the ipsilateral side in 3 cases (17.65%), 1 (5.9%) patient each had localised swelling and tenderness at the site of anomalous joint, bilateral fractures of the humeral neck and hand weakness. Involvement of the brachial plexus by the CCJ was described in six (35.29%) cases. Of these, three had a thoracic outlet syndrome and one case had bilateral brachial plexus involvement. Symptoms were ascribed to osteoarthritis of anomalous CCJ in three (17.65%) patients and supracoracoid impingement in three (17.65%) cases [1]. We present the case of a symptomatic osteoarthtritic CCJ in a 46-year-old male patient.

## Case report

**Figure 1.**
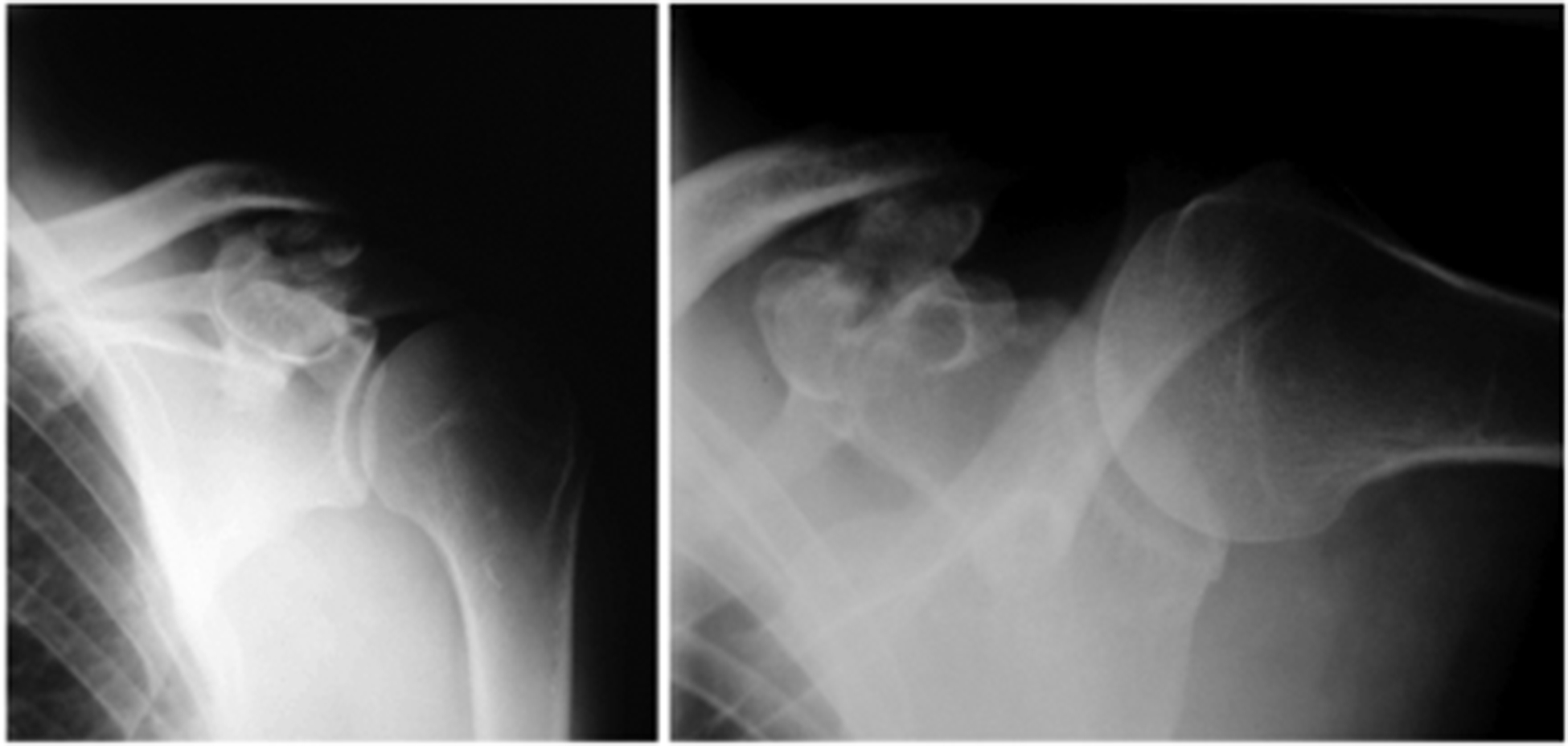
X-Ray of the left shoulder a.p. view a. and lateral view; b. shows osteoarthritis of a coracoclavicular joint with joint space narrowing, osteophytes and several loose bodies consistent (in the ap view).

**Figure 2.**
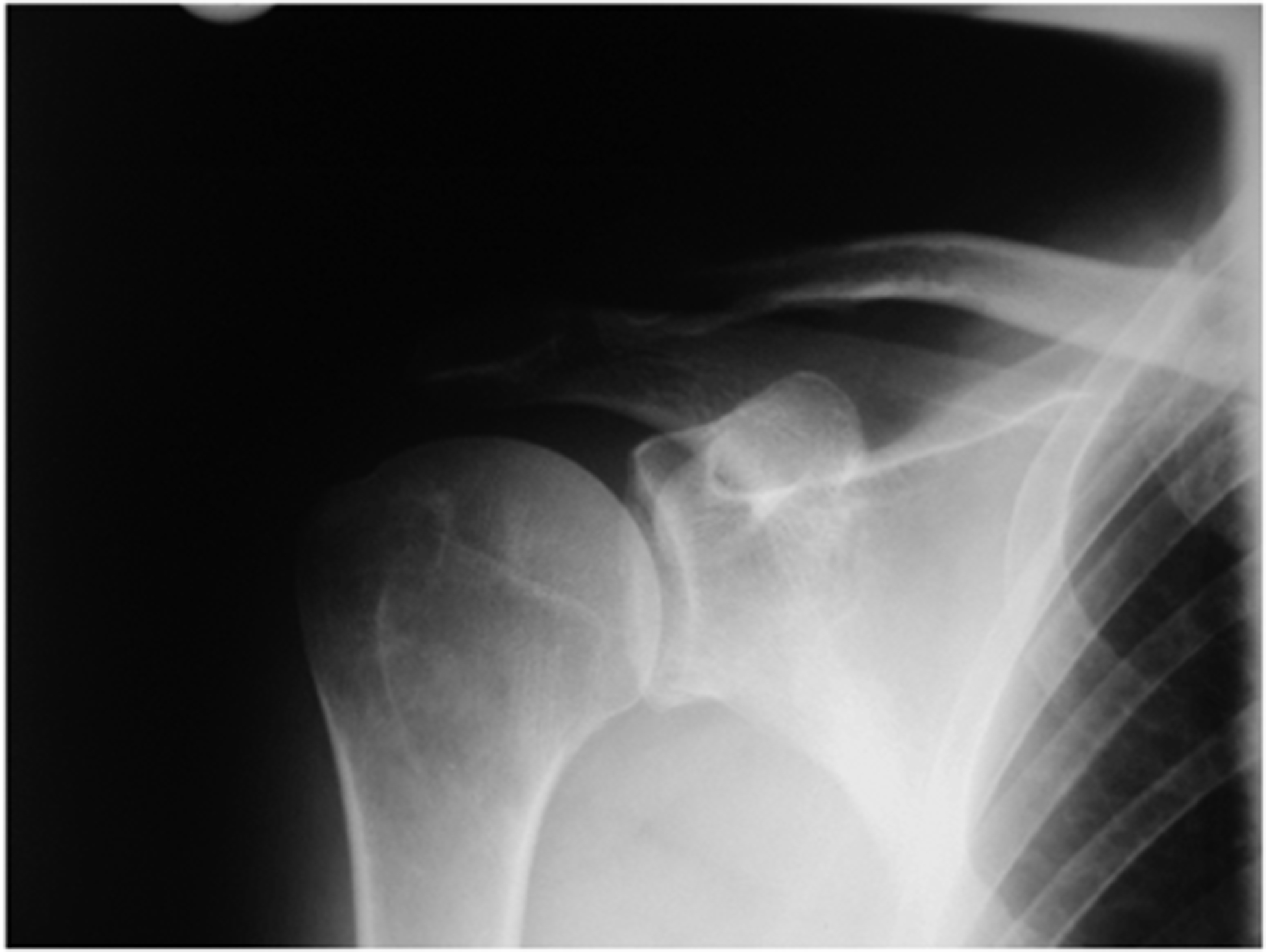
X-Ray of the right shoulder (a.p. view): No signs of a coracoclavicular joint.

A 46-year old male right-hand dominant caucasian patient presented in our outpatient department with increasing pain in his left shoulder over the past two years. There was no history of trauma or overuse concerning both shoulders. Clinical examination showed full active and passive range of motion, there was no local tenderness in the region of the greater tubercle and long biceps tendon or acromioclavicular joint. Pain was provoked by active abduction over 120°. There was local tenderness just above the left coracoid process. Radiographic examination of the left shoulder revealed the presence of a unilateral coracoclavicular joint with articular facets on the conoid tubercle of the clavicle and the superomedial surface of the coracoid process of the scapula with osteophytes, joint space narrowing and several possible loose bodies (Figure 1). The x-ray of the right shoulder showed no signs of CCJ (Figure 2). All symptoms immediately regressed after fluoroscopy guided intracapsular injection of a local anaesthetic (2 ml Xylocaine 2%) and corticosteroid (Prednisolon 10 mg). We recommended analgetic- antiphlogistic therapy (Ibuprofen 600 mg) on demand. At latest follow-up after 12 months the patient was nearly free of complaints. There was full ROM of both shoulders and slightly local tenderness just above the left coracoid process. We recommended another injection on demand and resection of the CCJ in case of recurrence if there was no sufficient pain relief after the second injection.

## Discussion

There are numerous pathological entities that may contribute to the development of a painful shoulder. In most of the cases, the typical signs and symptoms that are found together with the proper clinical and radiographic examination of the patient, contribute to relatively easy diagnosis [7]. The existence of a coracoclavicular joint is very uncommon. A degenerated coracoclavicular joint is even rarer [7]. In the case published by Cheung T *et al.* [8] there was a delay in correct therapy as the diagnosis was missed because of lack of knowledge of the possibility of such a finding. By reporting the actual case, we suggest that this pathological entity of a degenerated coracoclavicular joint should be kept in mind as a possible cause of shoulder pain. Clinical significance of CCJ remains uncertain. Hama *et al.* [9] reported a case of thoracic outlet syndrome that was caused by the existence of an irregular coracoclavicular joint. Nikolaides A *et al.* [7] were the first who published a symptomatic osteoarthritic CCJ. In this case symptomatic CCJ has been proved by immediate pain relief after intracapsular injection of local anaesthetic (Xylocaine 2%). According to Nehme *et al.* [1,6] the radiograph used to diagnose CCJ should be taken with the patient in a standing position, with both hands lying over the lateral aspect of each corresponding thigh [6]. In this position, the length of the conoid tubercle should be at least 3 mm, with either one or two sharp edges at the inferior aspect. Additional CT or MRI scans can helpful in unclear conditions. In these cases, Singh *et al.* [1] recommend dynamic MRI that not only gives detailed anatomy of the joint and nearby structures, but also highlights any undue traction/impingement of neurovascular structures in different arm positions, which may not be obvious in static MRI. Symptomatic CCJ has to be differentiated from subcoracoid impingement. Unlike subcoracoid impingement, where contact occurs between a laterally placed coracoid and the proximal humerus with flexion and internal rotation, the impingement in case of CCJ occurs in flexion and neutral rotation. Local anesthetics and steroids, injected in the CCJ, can differentiate the two entities. Faraj A [10] believes that coracoclavicular joints are congenital, and therefore often bilateral. In our case we found a unilateral CCJ. The average space between the clavicle and the coracoid process is normally 1.3 cm [10]. When this space is reduced, the upper part of the coracoid can impinge against the lower end of the clavicle projection during forward flexion. Excision of the joint in order to enlarge the space between the clavicle and the coracoid process is reported to be useful when non-operative treatment (physiotherapy and local steroid injection) fails [1,10,11].

The presence of a CCJ hampers normal movements of bones in the shoulder girdle, which are otherwise possible to a greater degree due to normal laxity of the coracoclavicular ligament complex. It is proposed that downwards pull on the coracoid process generated by the anomalous CCJ restricts the free upward movement of the acromion and leads to decreased space between the acromion and supraspinatus. This reduced functional space creates undue friction between these two structures, leading to impingement of the supraspinatus muscle [1,10,12].

Nonoperative interventions include anti-inflammatories, physiotherapy, lifestyle modification or local corticosteroid injection under fluoroscopy [13]. Operative intervention involves surgical excision of the joint [1,8,7,13].

We strictly follow Singh *et al.* [1] that nonsurgical treatment may be the treatment of choice in a select group of patients: the elderly, with low functional demand; patients with high American Society of Anesthesiologists (ASA) score who are high surgical risk and patients unwilling to undergo surgery. Although conservative treatment has shown low success rates in the literature, it should always be the first-line of treatment. Nikolaied published a case of conservative treatment of a symptomatic CCJ with a pain free period after 2 injections of 30 months. Failure of nonsurgical measures warrants operative excision of the joint, which has been described in open techniques whereas arthroscopic techniques could be possible, too.

## References

[1] Singh VK, Singh PK, Trehan R, Thompson S, Pandit R, (2011) Symptomatic coracoclavicular joint: incidence, clinical significance and available management options. Int Orthop 35: 1821–1826. 21761150 10.1007/s00264-011-1309-4 PMC3224626

[2] Gumina S, Salvatore M, De Santis R, Orsina L, Postacchini F (2002) Coracoclavicular joint: osteologic study of 1020 human clavicles. J Anat 201: 513–519. 12489763 10.1046/j.1469-7580.2002.00115.x PMC1570995

[3] Haramati N, Cook RA, Raphael B, McNamara TS, Staron RB, (1994) Coracoclavicular joint: normal variant in humans. A radiographic demonstration in the human and non-human primate. Skeletal Radiol 23: 117–119. 8191295 10.1007/BF00563205

[4] Kaur H, Jit I (1991) Brief communication: coracoclavicular joint in Northwest Indians. Am J Phys Anthropol 85: 457–460. 1928318 10.1002/ajpa.1330850409

[5] Moore RD, Renner RR (1957) Coracoclavicular joint. Am J Roentgenol 78: 86–88. 13435404

[6] Nehme A, Tricoire JL, Giordano G, Rouge D, Chiron P, (2004) Coracoclavicular joints. Reflections upon incidence, pathophysiology and etiology of the different forms. Surg Radiol Anat 26: 33–38. 14574466 10.1007/s00276-003-0178-y

[7] Nikolaides AP, Dermon AR, Papavasiliou KA, Kirkos JM (2006) Coracoclavicular joint degeneration, an unusual cause of painful shoulder: a case report. Acta Orthop Belg 72: 90–92. 16570902

[8] Cheung TF, Boerboom AL, Wolf RF, Diercks RL (2006) A symptomatic coracoclavicular joint. J Bone Joint Surg Br 88: 1519–1520. 17075101 10.1302/0301-620X.88B11.18198

[9] Hama H, Matsusue Y, Ito H, Yamamuro T (1993) Thoracic outlet syndrome associated with an anomalous coracoclavicular joint. A case report. J Bone Joint Surg Am 75: 1368–1369. 8408158 10.2106/00004623-199309000-00012

[10] Faraj AA (2003) Bilateral congenital coracoclavicular joint. Case report and review of the literature. Acta Orthop Belg 69: 552–554. 14748114

[11] Hall FJS (1950) Coracoclavicular joint: a rare condition treated successfully by operation. Br Med J 1: 766–768. 20787835 10.1136/bmj.1.4656.766 PMC2037114

[12] Singh VK, Singh PK, Balakrishnan SK (2009) Bilateral coracoclavicular joints as a rare cause of bilateral thoracic outlet syndrome and shoulder pain treated successfully by conservative means. Singapore Med J 50: e214–7. 19551300

[13] Ma FY, Pullen C (2006) A symptomatic coracoclavicular joint successfully treated by surgical excision. J Shoulder Elbow Surg 15: e1–4. 10.1016/j.jse.2005.05.008 16979041

